# Supplementary material for: Content-rich biological network constructed by mining PubMed abstracts
Source: BMC Bioinformatics. 2004 Oct 8;5:147. doi: 10.1186/1471-2105-5-147 (PMC528731; doi:10.1186/1471-2105-5-147)
Supplement: Additional File 2 — The original results of the above study (non-essential files are deleted to keep the file size under the limit set by BMC bioinformatics). [file 1471-2105-5-147-S2.bz2 › chilibotAdditionalFile2/dip10/471ID9660793E10527/html/CCL5_CXCR3.html]

 


 **CCL5** and **CXCR3** 
  
Found 237 abstracts in PubMed, retrieved 10.  
 

 What does Google say? 
 PDF only 
| .edu only 

---

**Interactive relationship** (e.g. stimulation, inhibition, etc)

**Neutral relationship**- The present findings suggest that MCP 1, RANTES  [ **CCL5** ] , and IP 10  [ **CXCR3** ]  may participate in the pathogenesis of mercuric chloride induced tubulointerstitial fibrosis with mononuclear cell infiltration, via CCR2, CCR1 or CCR5, and  **CXCR3** , respectively.  Ref: 12834626 Exp Mol Pathol, 2003
- DESIGN AND PATIENTS Immunocytochemistry and reverse transcriptase polymerase chain reaction RT PCR amplification were used to analyse the expression of monocyte chemoattractant protein MCP 1, RANTES  [ **CCL5** ]  regulated upon activation, normal T cell expressed and secreted, macrophage inflammatory protein MIP 1alpha, MIP 1beta, interferon IFN gamma inducible protein IP 10  [ **CXCR3** ]  and monokine induced by IFN gamma Mig in thyroid tissue from patients with Hashimoto s thyroiditis n = 4, Graves disease n = 6 and nonautoimmune multinodular goitre n = 4.  Ref: 12864798 Clin Endocrinol (Oxf), 2003

**Non-interactive relationship** (e.g. studied together, co-existance, homology, etc.)

- CONCLUSION We postulate that TFC may play a role in the pathogenesis of autoimmune thyroid disease as they are able to express the chemokines MIP 1alpha, MIP 1beta, MCP 1, RANTES  [ **CCL5** ] , IP 10  [ **CXCR3** ]  and Mig that would promote the infiltration of immune cells into the thyroid gland.  Ref: 12864798 Clin Endocrinol (Oxf), 2003
- RESULTS Expression of MCP 1, RANTES  [ **CCL5** ] , MIP 1alpha, MIP 1beta, IP 10  [ **CXCR3** ]  and Mig was demonstrated in all Hashimoto s and most Graves thyroid specimens but very little expression was detected in the nonautoimmune goitre samples.  Ref: 12864798 Clin Endocrinol (Oxf), 2003
- Infiltrating CD8 T Cells in Oral Lichen Planus Predominantly Express CCR5 and  **CXCR3**  and Carry Respective Chemokine Ligands RANTES  [ **CCL5** ]   **CCL5**  and IP 10  [ **CXCR3** ]  CXCL10 in Their Cytolytic Granules A Potential Self Recruiting Mechanism.  Ref: 12819030 Am J Pathol, 2003
- In the lesions, the expression of MCP 1 mRNA was most prominently elevated, and those of RANTES  [ **CCL5** ]  and IP 10  [ **CXCR3** ]  mRNAs also increased, and their proteins were localized in tubular epithelium.  Ref: 12834626 Exp Mol Pathol, 2003
- We investigated the potential down regulators of chemokine secretion IL 4, IL 10, IL 13, dexamethasone, and PGE2, IL 10 and IL 13 down regulate M. tuberculosis induced IL 8, IP 10  [ **CXCR3** ] , RANTES  [ **CCL5** ] , and MCP 1 secretion from MG 63 cells by between 48 and 94% P <.  Ref: 12797540 J Clin Immunol, 2003
- Furthermore, infiltrating T cells, particularly CD8 T cells, were positive for RANTES  [ **CCL5** ]   **CCL5**  and IP 10  [ **CXCR3** ]  CXCL10, the ligands of CCR5 and  **CXCR3** , respectively.  Ref: 12819030 Am J Pathol, 2003
- High levels of RANTES  [ **CCL5** ]   **CCL5** , MCP 1 CCL2, IP 10  [ **CXCR3** ]  CXCL10, and Mig CXCL9 simultaneously with mononuclear cell infiltration in the lungs was found.  Ref: 12875978 Am J Pathol, 2003
- Potential molecular differences between early and late rejection grade > =2 episodes were examined by testing chemokine and receptor gene expression.METHODS Expression of inducible protein IP 10  [ **CXCR3** ] , monokine induced by IFN gamma Mig, interferon inducible T cell alpha chemoattractant I TAC, regulated on activation normal T cell expressed and secreted RANTES  [ **CCL5** ] , and their receptors  **CXCR3**  and CCR5 was tested in 60 endomyocardial biopsies from 24 patients using quantitative Taqman real time polymerase chain reaction PCR.  Ref: 12829909 Transplantation, 2003
- Neuronal and astrocytic apoptosis in these cultures was significantly inhibited by co treatment with MCP 1 or RANTES  [ **CCL5** ]  but not IP 10  [ **CXCR3** ] .  Ref: 12753088 J Neurochem, 2003
- The biopsies were taken in the first 3 months or from the 9th to the 12th month following transplantation.RESULTS IP 10  [ **CXCR3** ] , Mig, RANTES  [ **CCL5** ] ,  **CXCR3** , and CCR5 expression levels were increased in the later versus earlier biopsies P< ; =0.01 despite no change in histologic rejection grade status.CONCLUSION These results demonstrate significantly increased expression of T cell chemoattractants in heart allografts during later rejection when compared with episodes occurring shortly after transplantation.  Ref: 12829909 Transplantation, 2003
- Furthermore, the presence of  **CCL5**  and CXCL10 in the cytolytic granules of tissue infiltrating CD8 T cells expressing CCR5 and  **CXCR3**  reveals a potential self recruiting mechanism involving activated effector cytotoxic T cells.  Ref: 12819030 Am J Pathol, 2003
- Despite there being no difference in expression of CC chemokine receptor 3  [ **CXCR3** ] , the priming effect of RANTES  [ **CCL5** ]  and eotaxin was significantly enhanced in eosinophils from the patients.  Ref: 12797483 Eur Respir J, 2003
